# Revolutionizing Addiction Medicine: The Role of Artificial Intelligence

**DOI:** 10.1192/j.eurpsy.2024.860

**Published:** 2024-08-27

**Authors:** S. Poudel, J. Choudhari, N. Panta, H. Kumar, D. Leszkowitz, S. S. Ahmed

**Affiliations:** ^1^Department of Research & Academic Affairs, Larkin Community Hospital, South Miami; ^2^Kathmandu Medical College, Kathmandu, United States; ^3^Dow University of Health Sciences, Karachi, Pakistan; ^4^Department of Addiction Medicine, Larkin Community Hospital, South Miami, United States

## Abstract

**Introduction:**

Addiction medicine is becoming more of an issue as addiction-related problems continue to plague people all over the globe, resulting in serious health consequences. Addiction has become increasingly prevalent in recent years, as have addiction-related disorders. For efficient care and improved patient outcomes, this growing pandemic requires early and precise identification. In the field of addiction medicine, artificial intelligence (AI) looks to be a feasible tool. This systematic review examines the current state of research on the use of AI in addiction medicine, including a variety of AI techniques, their efficiency compared to conventional diagnostic methods, and their potential influence on addiction therapy. While AI has great potential for transforming addiction treatment, further research is needed to assess its use fully.

**Objectives:**

The objective of this review is to assess the current state of research on the use of artificial intelligence in addiction medicine, focusing on its diagnostic efficacy and potential for revolutionizing addiction therapy.

**Methods:**

To evaluate the effectiveness of AI in addiction medicine, we conducted an extensive search of the PubMed database. Our search encompassed articles published in the English language from January 2013 to March 2023. Inclusion criteria encompassed studies reporting the utilization of AI for addiction diagnosis in human patients.

**Results:**

The initial PubMed search produced 100 papers, of which 15 were included after meticulous analysis and screening. These studies assessed diverse types of data, including patient records and behavioral patterns, employing various AI techniques, such as machine learning and deep learning. The findings indicate that AI can accurately and swiftly identify addiction-related issues, boasting high sensitivity and specificity rates. Additionally, AI demonstrates potential in identifying specific addiction subtypes and forecasting patient outcomes. Nevertheless, these studies also underscore certain limitations of AI, such as the requirement for extensive data and susceptibility to overfitting.

**Conclusions:**

Artificial intelligence holds the potential to revolutionize addiction medicine by enabling faster and more precise diagnostics, pinpointing specific addiction subtypes, and predicting patient outcomes. However, further research is imperative to validate AI’s efficacy across diverse patient populations and address challenges related to data accessibility, communication, and integration into clinical practice.

**Disclosure of Interest:**

None Declared

